# Trial of labor when twin B is slightly larger than twin A: A Danish prospective cohort study

**DOI:** 10.1002/ijgo.70246

**Published:** 2025-06-03

**Authors:** Mohammed R. Khalil, Diane Gorm Malberg‐Pedersen, Erling Andreasen, Sören Möller, Camilla Mirian Hartvigsen, Niels Uldbjerg

**Affiliations:** ^1^ Department of Obstetrics and Gynecology Lillebaelt University Hospital Kolding Denmark; ^2^ Research Unit OPEN, Department of Clinical Research University of Sothern Denmark and Odense University Hospital Odense Denmark; ^3^ Department of Obstetrics and Gynecology Odense University Hospital Odense Denmark; ^4^ Department of Obstetrics and Gynecology Aarhus University Hospital Aarhus Denmark; ^5^ Department of Clinical Medicine Aarhus University Aarhus Denmark

**Keywords:** birth weight discordance, emergency cesarean section, induction of labor, mixed‐mode twin birth, twin pregnancies

## Abstract

**Objectives:**

To assess whether a trial of labor at term in twin pregnancies, where twin B is at least 250 g heavier than twin A, is associated with increased risk of emergency cesarean section or mixed‐mode birth (vaginal delivery of twin A followed by cesarean of twin B). Existing guidelines on this topic are based on heterogeneous cohorts that combine planned cesarean, emergency cesarean, and mixed‐mode births, limiting direct conclusions on trial of labor outcomes.

**Methods:**

In this prospective cohort study, we included twin pregnancies referred to a Danish tertiary center from 2007 to 2019. Only those with gestational age ≥ 37 + 0 weeks and birth weight discordance <25% were eligible. Among 302 women undergoing trial of labor, 59 had a weight discordance ≥250 g with twin B as the larger fetus (“twin B larger”), while 243 formed the control group (“twins equal” or “twin A larger”). Primary outcomes were emergency cesarean section and mixed‐mode birth. Secondary outcomes included blood loss ≥1000 mL, Apgar score <7 at 5 min, umbilical artery pH <7.1, and neonatal length of stay.

**Results:**

The rate of emergency cesarean section was higher in the “twin B larger” group (29%) compared with controls (19%, *P* = 0.09). Mixed‐mode birth occurred in 8.5% of the “twin B larger” group and 11.5% of controls (risk difference, −3.0% [95% CI, −11.2% to 5.1%]). No significant differences were observed in secondary neonatal outcomes between groups.

**Conclusion:**

In twin pregnancies at term where twin B is slightly heavier than twin A, a trial of labor is not associated with a clinically meaningful increase in the risk of mixed‐mode birth. While the emergency cesarean section rate was numerically higher, this did not reach statistical significance and should not in itself contraindicate vaginal delivery. Further research with larger sample sizes is warranted to confirm these findings.

Regarding vaginal birth of twin pregnancies, the primary risk lies in delivering twin B.^1^ This becomes particularly worrisome when twin B outweighs twin A significantly, as such a weight discordance may escalate the risks of prolonged intervals between deliveries, head entrapment if twin B is not in a cephalic presentation, and the occurrence of mixed‐mode twin birth (where twin A is delivered vaginally and twin B abdominally). However, there is a lack of high‐quality data to establish a specific weight discordance threshold that would indicate a preference for planned abdominal birth over trial of vaginal birth. This explains the disparities seen among national guidelines.^1^ For instance, the National Institute for Health and Care Excellence (NICE) guideline from the United Kingdom recommends a 25% threshold,^2^ while the Danish guideline also utilizes a 25% threshold but advises to “consider abdominal birth”.^3^ In contrast, the US guidelines do not specify an upper weight discordance limit for attempting vaginal birth.^4^, ^5^ These guidelines note that it is relatively well established that twin pregnancies with a weight discordance (twin A or twin B being the larger one) between 30% and 40% at term of pregnancy are at increased risk of neonatal death after both abdominal (0.6%) and vaginal (0.8%) birth as compared with twin pregnancies without weight discordance (0.2%).^6^ However, weight discordance thresholds at the lower end of the spectrum (e.g. 250 g, or 25%) remain underexplored in the literature, despite the practical relevance of this information for clinical decision‐making.

Only one study has specifically examined weight discordance within the range of 250 g or 25%.^7^ Among the 73 twin pregnancies delivered vaginally where twin B exceeded twin A by 250 g, there was no increased risk of neonatal death (*n* = 3) or intraventricular hemorrhage (*n* = 0). However, this study did include preterm and very preterm deliveries, and it excluded pregnancies where both twins or twin B were delivered abdominally. Therefore, it did not address the specific outcomes related to “trial of labor,” including the associated risks of emergency cesarean section and mixed‐mode twin birth. These outcomes are crucial as they pose maternal and neonatal risks and can be emotionally distressing for parents, as well as resource‐intensive for maternity wards.

Thus, the aim of this study was to investigate the effects trial of labor in twin pregnancies at term, where twin B has a slight weight advantage over twin A (250 g or 25%), on the risks of emergency cesarean section and mixed‐mode twin birth within a Danish cohort. Our study focuses on this specific weight discordance range to fill the gap in understanding how such discordance may influence the likelihood of cesarean birth and the management of twin deliveries.

## 1 METHODS

This prospective cohort included all twin pregnancies referred to the Department of Obstetrics at Lillebælt University Hospital, Kolding, Denmark, from January 2007 to April 2019. Inclusion criteria were twin pregnancies with a gestational age at birth of ≥37 + 0 weeks and live fetuses on admission. Exclusion criteria were fetal anomalies at birth, twin‐twin transfusion syndrome, and birth weight discordance >25%. Weight discordance was calculated by dividing the inter‐twin weight difference by the smaller twin's birth weight.

Departmental policy during the study years encouraged a trial of vaginal birth in twins presenting with the first twin in a vertex position, regardless of the presentation and the weight of the second fetus. Breech extraction of the second twin was performed with the presence of an attending experienced physician.

The pregnancies were categorized into “B‐larger” (twin B ≥ twin A + 250 g), “equal” (twin B = twin A ± 249 g), and “A‐larger” (twin A ≥ twin B + 250 g). The primary outcomes were emergency cesarean section of both twins and mixed‐mode twin birth. The secondary outcomes were perinatal death, blood loss, vacuum‐assisted birth, Apgar score, umbilical artery pH, and length of nursery stay of neonate.

### 1.1 Statistical analysis

Quantitative data were first assessed for normality using the Shapiro–Wilk test (or other appropriate test if necessary). Descriptive statistics were used to summarize data (mean, standard deviation, median, interquartile range [IQR], and frequency distribution). Median and IQRs were compared using Kruskal‐Wallis test.

Categorical variables were compared using χ^2^ test or Fisher exact test if cells were expected to contain fewer than five patients. Continuous variables were compared using the Kruskal‐Wallis test for nonparametric data. *P* values <0.05 were considered statistically significant. All analyses were conducted using Stata version17 (StataCorp LLC).

### 1.2 Ethics

The data collection and storage for this project were approved by the Regional Ethical Authority “Region Syddanmark” under file number 18/53689.

## 2 RESULTS

A total of 481 twin pregnancies at term with birth weight discordance <25% were included. Among these, 96 pregnancies (20%) were classified as “twin B larger,” 234 (49%) as “twins equal,” and 151 (31%) as “twin A larger.” A planned trial of labor was documented in 302 pregnancies, of which 7.3% had a history of cesarean section. In comparison, 179 pregnancies were scheduled for planned cesarean section, and 19% of these women had previously undergone a cesarean. Maternal characteristics were comparable among the three groups, as shown in Table 1. Among those undergoing trial of labor, monochorionicity was more frequent in the “twin B larger” group (24%) than in the “twins equal” (14%) and “twin A larger” (12%) groups, although this difference was not statistically significant (*P* = 0.12) (Table 1).

**TABLE 1:** Maternal characteristics.

	Twin B larger	Twins equal	Twin A larger	*P* value
**Trial of labor, *n* = 302**	***n* = 59**	***n* = 151**	***n* = 92**	
Age (mean ± SD), years	32.2 ± 4.8	31.2 ± 4.6	31.4 ± 3.7	0.36
BMI ≥30, % (*n*), kg/m2	10% (6)	11% (16)	5% (5)	0.37
Nulliparous, % (*n*)	25% (15)	27% (41)	33% (30)	0.56
Monochorionic, % (*n*)	24% (14)	14% (21)	12% (11)	0.12
Assisted conception,^a^ % (*n*)	34% (20)	44% (67)	47% (43)	0.27
Prior cesarean birth, % (*n*)	5% (3)	7% (10)	10% (9)	0.56
Hypertensive disorders, % (*n*)	14% (8)	16% (24)	15% (14)	0.91
Gestational diabetes, % (*n*)	14% (8)	6% (9)	10% (9)	0.19
Labor induction, % (*n*)	47% (28)	52% (78)	57% (52)	0.54
**Planned CS, *n* = 179**	***n* = 37**	***n* = 83**	***n* = 59**	
Age, mean ± SD, y	32.9 ± 4.3	31.0 ± 4.6	32.0 ± 4.5	0.07
BMI ≥30, % (*n*), kg/m2	11% (4)	14% (12)	14% (8)	0.86
Nulliparous, % (*n*)	19% (7)	33% (27)	25% (15)	0.28
Monochorionic, % (*n*)	8% (3)	10% (8)	17% (10)	0.31
Assisted conception,^a^ % (*n*)	54% (20)	42% (35)	54% (32)	0.28
Prior cesarean birth, % (*n*)	24% (9)	18% (15)	17% (10)	0.64
Hypertensive disorders, % (*n*)	11% (4)	22% (18)	19% (11)	0.37
Gestational diabetes, % (n)	8% (3)	10% (8)	7% (4)	0.94

Abbreviations: BMI, body mass index (before pregnancy); CS, cesarean section; SD, standard deviation.

^a^ Assisted conception covers the range of medical procedures used to treat infertility.

None of the women scheduled for trial of labor underwent cesarean section before the onset of labor. The overall vaginal birth rate of both twins was 63% in the “twin B larger” group, 68% in the “twins equal” group, and 72% in the “twin A larger” group (*P* = 0.51) (Table 2). Emergency cesarean delivery of both twins occurred in 29%, 19%, and 20% of these groups, respectively (*P* = 0.24). The incidence of mixed‐mode birth—where twin A was delivered vaginally and twin B by cesarean—was 8% in the “twin B larger” group, 13% in the “twins equal” group, and 9% in the “twin A larger” group (*P* = 0.43). Regarding maternal complications, postpartum blood loss ≥1000 mL was recorded in 12% of the “twin B larger” group, 8% in the “twins equal” group, and 5% in the “twin A larger” group (*P* = 0.21). Blood loss was reported as a secondary outcome in accordance with the study protocol.

**TABLE 2:** Maternal outcomes.

	Twin B larger	Twins equal	Twin A larger	*P* value
**Trial of labor, *n* = 302**	***n* = 59**	***n* = 151**	***n* = 92**	
Vaginal birth of both twins, % (*n*)	63% (37)	68% (103)	72% (66)	0.51
Emergency CS (exclusive mixed), % (*n*)	29% (17)	19% (28)	20% (18)	0.24
Twin B born by emergency CS, % (*n*)	8% (5)	13% (20)	9% (8)	0.43
Blood loss including reoperation ≥1000 mL, % (*n*)	12% (7)	8% (12)	5% (5)	0.21
Vacuum‐assisted birth (twin A or B), % (*n*)	10% (6)	11% (16)	10% (9)	0.98
**Planed CS, *n* = 179**	***n* = 37**	***n* = 83**	***n* = 59**	
As planned CS, % (*n*)	86% (32)	77% (64)	92% (54)	0.06
Vaginal birth, % (*n*)	0% (0)	0% (0)	0% (0)	–
Emergency CS, % (*n*)	14% (5)	23% (19)	8% (5)	0.06
Twin B born by emergency CS, % (*n*)	0% (0)	0% (0)	0% (0)	–
Blood loss including reoperation ≥1000 mL, % (*n*)	19% (7)	10% (8)	17% (10)	0.33
Vacuum‐assisted birth (twin A or B), % (*n*)	–	–	–	–

Abbreviation: CS, cesarean section.

Neonatal outcomes are summarized in Table 3. One woman in the “twin A larger” group experienced perinatal loss of both twins during a trial of labor. Among the 604 neonates delivered following a trial of labor, 10 (1.7%) had a 5‐min Apgar score <7, while only one (0.3%) was recorded among the 358 neonates delivered via planned cesarean. No cases of umbilical artery pH <7.1 were observed in either group. Respiratory distress syndrome (RDS) occurred in 108 neonates (18%) born after trial of labor and 59 (16%) after planned cesarean section.

**TABLE 3:** Neonatal outcomes.

	Twin B larger	Twins equal	Twin A larger	*P* value
**Trial of labor, *n* = 302**	**Pairs: 59** **Individuals: 118** **(19.5%)**	**Pairs: 151** **Individuals:** **(50.0%)**	**Pairs: 92** **Individuals:** **(30.5%)**	
Twin A birth weight, mean ± SD, g	2613 ± 302	2932 ± 1042	2989 ± 280	0.00
Twin B birth weight, mean ± SD, g	3125 ± 329	2933 ± 1044	2482 ± 296	0.00
Twin A IUGR, % (*n*)	38 (64.4)	47 (31.1)	7 (7.6)	0.00
Twin B IUGR, % (*n*)	4 (6,8)	45 (29.8)	70 (76.1)	0.00
Twin A female sex, % (*n*)	53% (31)	42% (64)	36% (33)	0.20
Twin B female sex, %(n)	42% (25)	49% (74)	61% (56)	0.10
Twin A Apgar score <7 at 5 min,^a^ % (*n*)	3% (2)	0% (0)	4% (4)	0.16
Twin B Apgar score <7 at 5 min,^a^ % (*n*)	2% (1)	2% (3)	3% (3)	0.88
Twin A pH <7.1,^b^ % (*n*)	0% (0)	0% (0)	0% (0)	–
Twin B pH <7.1,^b^ % (*n*)	0% (0)	0% (0)	0% (0)	–
Twin A pH <7.0,^b^ % (*n*)	0% (0)	0% (0)	0% (0)	–
Twin B pH <7.0,^b^ % (*n*)	0% (0)	0% (0)	0% (0)	–
Twin A hyperbilirubinemia, % (*n*)	4 (6.8)	26 (17.2)	17 (18.5)	0.50
Twin B hyperbilirubinemia, % (*n*)	7 (11.9)	26 (17.2)	15 (16.3)	0.50
Twin A RDS, % (*n*)	8 (13.6)	15 (9.9)	13 (14.1)	0.65
Twin B RDS, % (*n*)	15 (25.4)	35 (23.2)	22 (23.9)	0.94
Twin A death, % (*n*)	0% (0)	0% (0)	1% (1)	0.50
Twin B death, % (*n*)	0% (0)	0% (0)	1% (1)	0.50
Postnatal ward, mean ± SD, days	6.9 ± 4.2	6.5 ± 3.2	7.5 ± 3.4	0.03
Presentation				
Twin A vertex, % (*n*)	55 (93.2)	142 (94.0)	85 (92.4)	0.83
Twin B vertex, % (*n*)	37 (62.7)	82 (54.3)	53 (57.6)	0.55
Twin A breech, % (*n*)	0 (0.0)	1 (0.7)	0 (0.0)	1.00
Twin B breech, % (*n*)	9 (15.3)	43 (28.5)	27 (29.3)	0.10
**Planed CS, *n* = 179**	**Pairs: *n* = 37** **Individuals:** **(20.7%)**	**Pairs: *n* = 83** **Individuals:** **(46.4%)**	**Pairs: *n* = 59** **Individuals:** **(33.0%)**	
Twin A birth weight, mean ± SD, g	2560 ± 280	2946 ± 1156	3016 ± 325	0.00
Twin B, birth weight, mean ± SD, g	3052 ± 330	2963 ± 1153	2507 ± 321	0.00
Twin A IUGR, % (*n*)	23 (62.2)	30 (36.1)	8 (13.6)	0.00
Twin B IUGR, % (*n*)	5 (13.5)	32 (38.6)	42 (71.2)	0.00
Twin A female sex, % (*n*)	76% (28)	55% (46)	49% (29)	0.04
Twin B female sex, % (*n*)	41% (15)	55% (46)	58% (34)	0.26
Twin A Apgar score <7 at 5 min,^a^ % (*n*)	0% (0)	1% (1)	0% (0)	1.00
Twin B Apgar score <7 at 5 min,^a^ % (*n*)	0% (0)	0% (0)	0% (0)	–
Twin A pH <7.1,^b^ % (*n*)	0% (0)	0% (0)	0% (0)	–
Twin B pH <7.1,^b^ % (*n*)	0% (0)	0% (0)	0% (0)	–
Twin A pH <7.0,^b^ % (*n*)	0% (0)	0% (0)	0% (0)	–
Twin B pH <7.0,^b^ % (*n*)	0% (0)	0% (0)	0% (0)	–
Twin A hyperbilirubinemia, % (*n*)	5 (13.5)	12 (14.5)	8 (13.6)	1.00
Twin B hyperbilirubinemia, % (*n*)	6 (16.2)	11 (13.3)	8 (13.6)	0.92
Twin A RDS, % (*n*)	8 (21.6)	10 (12.0)	6 (10.2)	0.27
Twin B RDS, % (*n*)	8 (21.6)	11 (13.3)	16 (27.1)	0.12
Twin A death, % (*n*)	0 (0.0)	0 (0.0)	0 (0.0)	–
Twin B death, % (*n*)	0 (0.0)	0 (0.0)	0 (0.0)	–
Postnatal ward, mean ± SD, d	5.6 ± 2.2	7.0 ± 10.6	6.8 ± 2.4	0.00

*Note*: Total indicates *V*_*A*_ ≥ *V*_*B*_ + 250 g and *V*_*A*_ = *V*_*B*_ ± 249 g.

Abbreviations: IUGR, intrauterine growth restriction; RDS, respiratory distress syndrome; SD, standard deviation.

^a^ Data are presented as mean values.

^b^ Data are presented as measurements from umbilical cord blood just after birth (<1 min).

When stratified by group, the RDS rate in the “twin B larger” group was 19% (23 of 118 neonates), affecting eight twin As and 15 twin Bs. In the “twins equal” group, RDS was diagnosed in 50 of 302 neonates (17%), with 15 cases in twin A and 35 in twin B. Similarly, in the “twin A larger” group, RDS occurred in 35 of 184 neonates (19%), with 13 cases in twin A and 22 in twin B.

Additionally, the birth weight differences between twins were compared among groups. The “twin B larger” group had a median inter‐twin weight difference of 320 g (IQR, 260–370 g), the “twins equal” group had 90 g (IQR, 30–150 g), and the “twin A larger” group had 310 g (IQR, 260–360 g). This difference was statistically significant (*P* < 0.001) (Table 3).

## 3 DISCUSSION

This prospective cohort study on trial of labor in twin pregnancies included 59 cases where twin B was at least 250 g heavier than twin A, but with a birth weight discordance <25%. This range was deliberately selected to reflect a subtle but clinically relevant weight difference, in alignment with guidelines that consider 25% discordance a threshold for concern.^1^, ^2^, ^3^

Within the “twin B larger” group, the mixed‐mode birth rate (9%) was not significantly higher than in the “twins equal” group (13%, *P* = 0.43). However, due to the limited sample size, this lack of statistical significance must be interpreted cautiously. It is possible that a larger cohort could reveal a significant difference. Therefore, while the findings are reassuring, they are not conclusive. The limited statistical power of the study is a key limitation, and no definitive claims can be made about safety or risk based solely on our data.

A notable strength of the study is that all clinical decisions and data entries were performed and recorded prospectively by a single experienced obstetrician (E.A.). This ensured consistency in data collection and minimized classification bias between trials of labor and planned cesarean delivery. The high rate of women who underwent trial of labor (63%) despite some being clinically ineligible mirrors or exceeds rates reported in other cohorts, such as 71% in a Finnish study^2^ and 39% in a US cohort,^3^ supporting the internal and external validity of our findings.

Nevertheless, the study was not adequately powered to explore important clinical modifiers, such as chorionicity,^4^ parity,^2^ noncephalic presentation of twin B,^2^ or the role of estimated fetal weight before labor onset.^5^ Future studies should account for these potential confounders to refine counseling and decision‐making for twin births.

Interestingly, we observed that in 61% of weight‐discordant pairs, the larger twin was the presenting twin. This is consistent with previous literature. Usta et al.^6^ reported that 56% of the larger twins were the presenting twin and suggested a potential link to gestational age. It also aligns with findings from several studies noting that second‐born twins are typically smaller than their co‐twin at birth.^7^, ^8^, ^9^ The biological and positional mechanisms behind this distribution remain unclear, but may have clinical relevance in predicting labor outcomes.

Our findings did not support the initial hypothesis that the mixed‐mode birth rate would be higher in cases where twin B was heavier. In fact, the rate in the “twin B larger” group (9%) was comparable to that in the “twins equal” group (13%) and only marginally higher than the 6% mixed‐mode rate in the Finnish cohort,^2^ which also included late preterm deliveries (>35 + 0 weeks), and the 4% in the Twin Birth Study, which included births from >32 + 0 weeks.^10^ More surprisingly, the rate of cesarean delivery of both twins was highest in the “twin B larger” group (29%), compared with 19% in the “twins equal” and “twin A larger” groups (*P* = 0.09). Among women undergoing trial of labor in the “twin B larger” group, 63% still achieved vaginal delivery of both twins.

In terms of neonatal outcomes, acidosis was rare. No neonate had an umbilical artery pH <7.1, including twin Bs. This was more favorable than findings from the Finnish study, where the pH <7.05 rate was 5% for twin B after vaginal birth.^2^ Similarly, the Twin Birth Study reported <1% of neonates with Apgar scores <4 at 5 min.^10^ Rates of RDS in our cohort were higher: 19% in the “twin B larger” group and 17% in the pooled comparator groups. These rates were higher than those reported by Usta et al.,^6^ who found 5.5% and 9.9% for comparable groups, respectively.

Consistent with previous literature, our study observed a trend toward fewer adverse neonatal outcomes following planned cesarean delivery, in line with Zafarmand et al.^10^ However, while their study showed a higher perinatal death rate for both twins in the vaginal birth group (four for twin A and five for twin B), our cohort had only one perinatal death, observed in the “twin A larger” group. Notably, we did not find a higher incidence of adverse outcomes for twin Bs compared with twin As, possibly because our inclusion criteria excluded cases with weight discordance >25%, where risks may be more pronounced.^10^

Finally, from a clinical perspective, when a woman with twins at ≥37 + 0 weeks of gestation presents with a modest weight discordance (i.e., <25%) where twin B is larger, these data do not indicate a need for planned cesarean section solely based on the weight difference. However, the decision should be informed by full counseling about potential risks, acknowledging the study's limitations. Where cesarean is chosen under such circumstances, it remains debatable whether the indication should be recorded as a maternal request or clinical recommendation.

## 4 CONCLUSION

Our results suggest that modest inter‐twin weight discordance alone, even when twin B is the larger fetus, should not be considered a contraindication to vaginal delivery at term.

## AUTHOR CONTRIBUTIONS

All authors contributed equally to the manuscript.

## CONFLICT OF INTEREST STATEMENT

The authors report no conflicts of interest.

## Data Availability

Research data are not shared.
